# Loop-Mediated Isothermal Amplification Assay for the Detection of Citrus Canker Causing Bacterial Variant, *Xanthomonas citri* pv. *citri* A^w^ Strain

**DOI:** 10.3390/ijms252111590

**Published:** 2024-10-29

**Authors:** Sree Harsha Sidireddi, Jong-Won Park, Marissa Gonzalez, Mamoudou Sétamou, Madhurababu Kunta

**Affiliations:** Citrus Center, Texas A&M University Kingsville, Weslaco, TX 78599, USA; harshasidireddi60@gmail.com (S.H.S.); marissa.gonzalez@tamuk.edu (M.G.); mamoudou.setamou@tamuk.edu (M.S.)

**Keywords:** citrus canker, *Xanthomonas citri* pv. *citri*, *Xcc* A, *Xcc* A^w^, lateral flow immunoassay, *avrGF1* gene, *pthA* gene

## Abstract

Citrus canker, a highly transmissible bacterial disease, has three major types, with Asiatic canker (Canker A), caused by *Xanthomonas citri* pv. *citri* (*Xcc* A), being the most widespread and severe, affecting most citrus varieties. *Xcc* A has two mild variants, *Xcc* A* and A^w^ with a limited host range, reported in Southwest Asia and Florida, respectively. Since 2015, the canker caused by *Xcc* A^w^ has been being reported in the Rio Grande Valley of South Texas where the Texas commercial citrus industry is located. In 2016, a more severe Canker A was reported in the upper Texas gulf coast region, north of the Rio Grande Valley, posing a potential threat to the Texas citrus industry. Given that existing diagnostic methods cannot reliably distinguish *Xcc* A^w^ from *Xcc* A, we developed a loop-mediated isothermal amplification (LAMP) assay specific to *Xcc* A^w^ (LAMP-A^w^) for rapid, field-based identification of this bacterial variant. The detection limit of LAMP-A^w^ was ~4.52 Log_10_ copies of the target molecule. This study also evaluated the field applicability of the LAMP-A^w^ assay by coupling the LAMP-A^w^ assay with a lateral flow immunoassay system.

## 1. Introduction

Citrus bacterial canker is caused by *Xanthomonas citri*, which caused severe economic losses in citrus industries in the Gulf Coast states of the United States after its introduction from Japan in the early 1900s [1,2]. Citrus canker was also reported in Australia in 1912, South Africa in 1916 and Brazil in 1957, which is now reported in over 30 different countries [2,3,4,5]. Although the eradication program that was deployed in the U.S. Gulf States in the early to mid-1900s was successful [6], the disease re-emerged in Florida in the 1980s and 1990s, which led to the re-establishment of a state-wide quarantine program, resulting in the removal of ~1.56 million commercial trees [7,8,9]. However, the eradication program was dismissed in 2006 as the canker became endemic in Florida in the early 2000s [10,11]. Similarly, the eradication program in other countries was not successful. In Australia, the citrus canker reappeared in 1991 and again in 2004 [5,12]. It was estimated that Brazil spent approximately USD 116 million over 10 years to remove the infected trees, but the disease is still present in Brazil [13,14].

There are three major types of citrus canker, each of which is caused by a different pathovar of *X. citri*; Asiatic canker (Canker A) is caused by *X. citri* pv. *citri* (*Xcc* A), and Cancrosis B (Canker B) and Cancrosis C (Canker C) are caused by *X. citri* pv. *aurantifolii* pathotype B (*Xau* B) and C (*Xau* C), respectively [15]. Among them, Canker A is the most widespread and severe type of citrus canker affecting most citrus varieties [1,2]. Thus far, two variants, A* and A^w^, of *Xcc* A have been identified, respectively, in Southwest Asia and Florida [16,17]. Both *Xcc* A* and A^w^ have a narrow host range known to be limited to Mexican limes (*Citrus aurantifolii*) for *Xcc* A* and Mexican limes and alemow (*C. macrophylla* Wester) for *Xcc* A^w^ [16,17]. In Texas, the incidence of *Xcc* A^w^ was reported in residential Mexican lime trees in Lower Rio Grande Valley in 2015 [18]. Later, the citrus canker caused by *Xcc* A was confirmed in the Houston area in 2016 [19]. Although the incidence of *Xcc* A has been thus far limited to the Greater Houston area, the occurrence of Canker A in this region has posed a potential threat to the Texas commercial citrus industry located in the Rio Grande Valley of South Texas.

Since 2015, *Xcc* A^w^ incidence has been sporadically, but continuously, reported in the Rio Grande Valley, resulting in the expansion of the quarantine areas in the valley (https://texasagriculture.gov/Regulatory-Programs/Plant-Quality/Pest-and-Disease-Alerts, accessed on 24 October 2024). Due to the geographical proximity between Greater Houston area and the Rio Grande Valley of South Texas, when new outbreak of citrus canker is reported in the Rio Grande Valley, it is necessary to quickly determine whether the new canker case is caused by *Xcc* A or A^w^ to deploy proper disease management practices as early as possible to prevent the severe canker type A from spreading. However, the current canker diagnostic methods based on qPCR or immunoassay cannot detect *Xcc* A^w^ [20,21,22,23,24,25,26,27].

In contrast to PCR or qPCR, which require specialized laboratory equipment, isothermal DNA amplification methods, like loop-mediated isothermal amplification (LAMP), can be performed at a constant temperature without a thermal cycler, making them practical alternatives since their introduction in the 1990s [28,29]. Major isothermal amplification methods include loop-mediated isothermal amplification (LAMP) [30], recombinase polymerase amplification (RPA) [31], nucleic acid sequence-based amplification (NASBA) [32], and helicase-dependent amplification (HDA) [33]. Among these isothermal methods, this study adopted the LAMP technique to develop a LAMP assay specific to *Xcc* A^w^ (LAMP-A^w^) mainly due to the ease of access to this technique and required reagents. In this study, the LAMP primers specific to *Xcc* A^w^ were developed based on the *avrGF1* gene, an avirulence gene of the *Xcc* A^w^ strain, which causes a hypersensitive reaction (HR) in grapefruit [34]. The study evaluated the specificity of the LAMP assay towards *Xcc* A^w^ and its field applicability by combining the assay with a lateral flow immunoassay system.

## 2. Results

### 2.1. Detection Limit of the LAMP Assay Specific to Xcc A^w^

**Figure 1:**
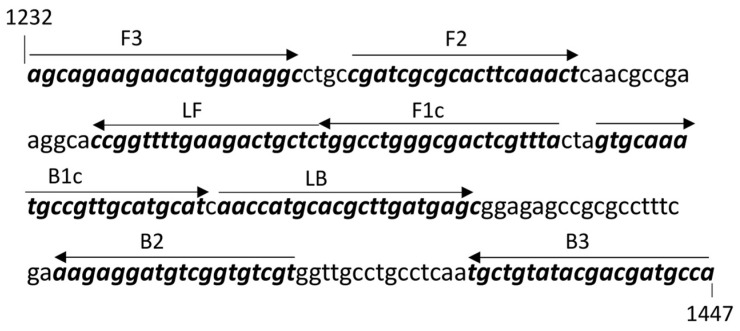
LAMP primers designed based on the *avrGF1* gene (DQ275469.1) of *Xanthomonas citri* pv. *citri* A^w^ strain. The primer sequences were highlighted in bold, and their names were indicated above the arrow. The arrow indicated the orientation of the LAMP primers. Two internal primers were designed to target two regions, F1c and F2 for FIP, and B1c and B2 for BIP. The nucleotide position numbers in the figure denote the position of the LAMP target in the *avrGF1* gene.

After designing a set of LAMP primers based on the *avrGF1* gene of *Xcc* A^w^ (DQ275469.1) (Figure 1), the LAMP-A^w^ assay was optimized on a CFX96 real-time system (BioRad) using *Bst* 2.0 WarmStart DNA polymerase (NEB) with the crude DNA extracts prepared from *Xcc* A^w^ positive leaf samples by soaking the canker lesion in the nuclease-free water followed by boiling for 10 min. The LAMP-A^w^ amplification on the real-time system was monitored every 30 s (i.e., One LAMP-A^w^ cycle = 30 s) for up to 90 LAMP cycles. The LAMP protocol for the *Bst* 2.0 DNA polymerase was used as a guideline for the optimization of the LAMP-A^w^ assay, from which 68 °C, 5.5 µM MgSO_4_ and the primer molar ratio of 1.6 µM (FIP/BIP):0.4 µM (LF/LB) and 0.8 µM (F3/B3) were determined as an optimum for LAMP-A^w^ reaction.

**Figure 2:**
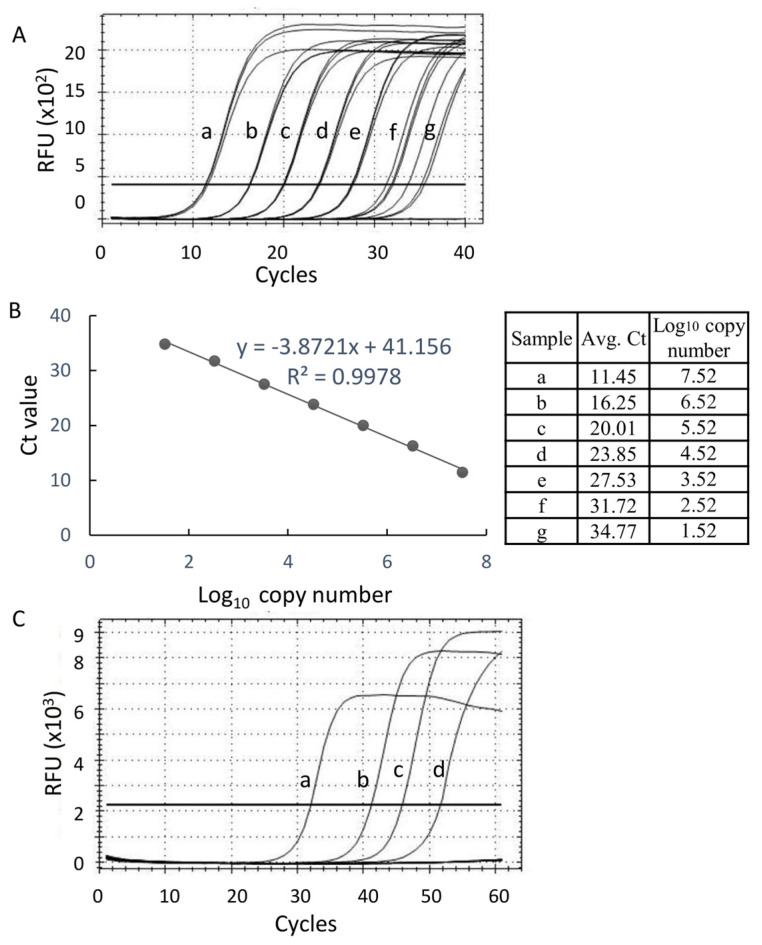
Determination of the detection limit of LAMP-A^w^ assay using a serially diluted recombinant plasmid containing the LAMP target (F3-B3 PCR amplicon). (**A**) Real-time PCR (qPCR) amplification curve conducted with LAMP F3/B3 primer set using a serially diluted recombinant plasmid as a template. (**B**) Standard curve generated with the qPCR results in (**A**). The table on the right shows the average qPCR Ct value of triplicates in (**A**) and the Log_10_ copy number of the target molecule of each sample that was calculated using a linear regression equation of the standard curve; y = −3.8721x + 41.161 (R^2^ = 0.9978) where y and x denote Ct and the Log_10_ target copy number, respectively. (**C**) The amplification curve of the LAMP-A^w^ assay was conducted with the serially diluted recombinant plasmid DNA that was used in (**A**). The relative fluorescence unit (RFU) of the LAMP reaction in (**C**) was measured every 30 s (i.e., one LAMP cycle = 30 s). The sample IDs are indicated in the figure. The threshold lines for qPCR and LAMP-A^w^ are indicated in the amplification graphs in (**A**) and (**C**), respectively.

In order to estimate the detection limit of the LAMP-A^w^ assay, recombinant plasmid DNA containing the PCR amplicon obtained with the primer set, F3/B3 (Figure 1), were constructed, from which a series of 10-fold dilution was prepared to generate a standard curve based on the qPCR results (Figure 2A,B). According to the data obtained with the LAMP-A^w^ reaction using the serially diluted recombinant plasmid DNA, the detection limit of LAMP-A^w^ was estimated to be 4.52 Log_10_ copies of the target molecules in the reaction (Figure 2C).

### 2.2. Specificity of the LAMP-A^w^ Assay

**Figure 3:**
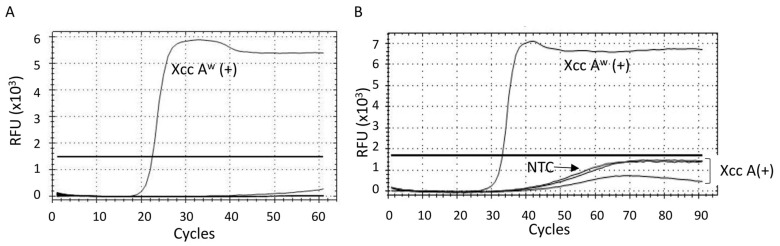
The specificity of the LAMP-A^w^ assay using (**A**) DNA fractions prepared from *Candidatus* Liberibacter asiaticus (CLas)-positive and canker-negative leaf samples collected in the field and (**B**) two *Xcc* A positives (Xcc A(+)) and one *Xcc* A^w^ positive DNA sample (Xcc A^w^(+)). The non-template control (NTC) is indicated in the figure. The threshold line was indicated in the graph. The RFU of the LAMP-A^w^ reaction was monitored every 30 s (i.e., one LAMP cycle = 30 s).

The specificity of LAMP-A^w^ assay was determined using the DNA extracts prepared from *Candidatus* Liberibacter asiaticus (CLas)-positive grapefruit leaves and field samples that tested negative for citrus canker by qPCR. The data showed no cross-reactivity of the LAMP-A^w^ assay with the CLas-positive and *Xcc*-negative samples (Figure 3A). In addition, the *Xcc* A^w^ strain specificity of the LAMP-A^w^ assay was examined against two *Xcc* A-positive DNA fractions, which confirmed that the LAMP-A^w^ assay had no cross-reactivity with *Xcc* A (Figure 3B).

### 2.3. Detection Efficiency of LAMP-A^w^ Assay

The detection efficiency of LAMP-A^w^ assay was examined using 19 field samples (14 leaves with canker lesions and 5 non-symptomatic leaves) by comparing the LAMP-A^w^ result with the qPCR data obtained from the F3/B3 primer set (Figure 1). In addition, since a LAMP assay, CBC-LAMP that was developed by Rigano et al. [24], is available for *Xcc* detection by targeting the *pthA* gene of *Xcc*, the CBC-LAMP assay was also included in the analysis for comparison. Among 19 samples tested in the study, 14 samples tested positive for *Xcc* by qPCR and CBC-LAMP, respectively (Table 1). These 14 samples also tested positive by the LAMP-A^w^ assay (Table 1). These data indicated that the detection efficiency of the LAMP-A^w^ assay using canker symptomatic leaves was comparable to that of qPCR and CBC-LAMP.

### 2.4. Field Applicability of LAMP-A^w^ Coupled with Lateral Flow Assay

**Figure 4:**
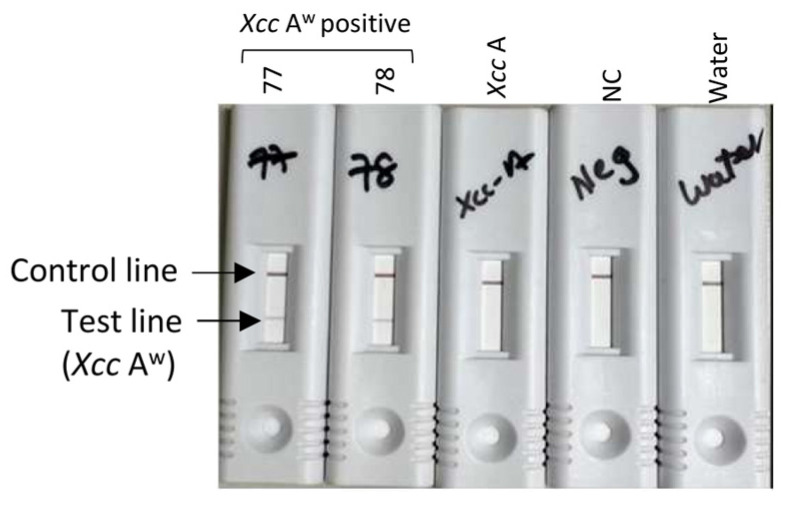
Evaluation of the specificity of LAMP-A^w^ assay coupled with lateral flow immunoassay. Sample IDs are shown on top. Samples 77 and 78 were prepared from *Xcc* A^w^-positive leaf samples. *Xcc* A is the DNA fraction prepared from a *Xcc* A positive sample. NC (negative control) was the healthy leaf DNA fraction. The ‘water’ sample indicates the LAMP reaction conducted with nuclease-free water. The control and test lines are indicated on the left, which detected the gold conjugate and Biotin- and DIG-labeled LAMP-A^w^ products, respectively.

In order to examine the field applicability of the LAMP-A^w^ assay, the LAMP-A^w^ reaction was conducted with crude DNA extracts prepared with 0.8% NaOH or AMP1 buffer (Agdia) following the manufacturer’s instructions, where the sample boiling step is not required for the crude DNA extract preparation. In addition, the 5′ end of two loop primers, LF and LB (Figure 1), were labeled with Biotin and DIG, respectively, to enable the visual confirmation of the LAMP-A^w^ test result on the lateral flow immunoassay cassette. Figure 4 showed that the LAMP-A^w^ assay coupled with the lateral flow immunoassay system successfully detected two *Xcc* A^w^ positive samples without cross-reactivity with the *Xcc* A positive sample.

**Figure 5:**
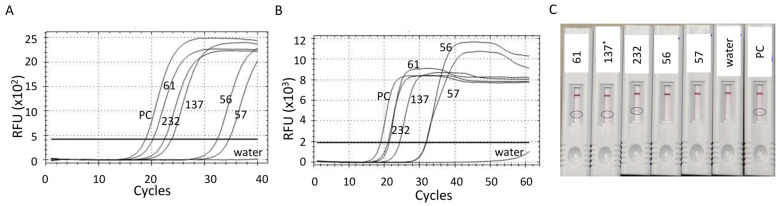
Estimation of the efficiency of the LAMP-A^w^ assay coupled with a lateral flow immunoassay system using samples with various *Xcc* A^w^ titers based on qPCR Ct values (**A**). (**B**) The LAMP-A^w^ assay conducted with the samples shown in (**A**) on a real-time PCR instrument. (**C**) The LAMP-A^w^ results tested on the lateral flow immunoassay system. The sample IDs are indicated in the figure. PC denotes the *Xcc* A^w^ positive control. Water is a non-template control of the LAMP-A^w^ reaction conducted with nuclease-free water. The oval in (**C**) indicated a positive signal on the test line of the lateral flow immunoassay cassette. * The band intensity of 137 on the test line of the lateral flow immunoassay cassette was faint. The RFU of the LAMP-A^w^ reaction was monitored every 30 s (i.e., one LAMP-A^w^ cycle = 30 s).

To estimate the efficiency of the lateral flow immuno-detection system, five *Xcc* A^w^-positive crude DNA extracts, prepared with 0.8% NaOH, which had variable *Xcc* titers, were selected. Due to the inhibitory effect of 0.8% NaOH on the LAMP-A^w^ reaction, the LAMP reaction in this experiment was conducted with 5 µL of 1/10 diluted crude DNA extracts with nuclease-free water. In order to compare the results obtained with the lateral flow immunoassay cassette, both LAMP-A^w^ and qPCR assays were also conducted on a CFX real-time system using 5 µL and 1 µL of 1/10 diluted crude DNA extracts, respectively. The data showed that while all five *Xcc* A^w^-positive crude extracts tested positive by both qPCR and LAMP-A^w^ assays conducted on the real-time system (Figure 5A,B), only three *Xcc* A^w^-positive crude extracts that had higher *Xcc* A^w^ titer tested positive on the lateral flow immunoassay cassette (Figure 5C). These data indicated that the lateral flow immunoassay system is less sensitive than the LAMP-A^w^ assay conducted on a real-time PCR instrument. Although LAMP-A^w^ coupled with a lateral flow assay is less sensitive than the one on a real-time system, the data also showed that the LAMP-A^w^ assay coupled with the lateral flow immunoassay system can be used in the field without the need for a real-time PCR system using crude DNA extracts. However, the data clearly indicated that any canker sample that tested negative for *Xcc* A^w^ on the lateral flow assay system needs to be retested in the lab on a real-time PCR instrument.

## 3. Discussion

Due to the re-emergence of citrus canker in Florida where the citrus canker became endemic [2,11], Texas initiated a state-wide citrus canker survey beginning in 1999, from which the incidence of the canker case caused by *Xcc* A^w^ in the Lower Rio Grande Valley of South Texas and a more severe Canker A in the Texas Upper Gulf Coast region was confirmed in 2015 and 2016, respectively [18,19]. Although thus far Canker A incidence is limited to Houston and nearby counties (the Greater Houston area) [19], the continued incidence of Canker A in the Greater Houston area has put commercial citrus growers in Texas on alert in the Mid and Lower Rio Grande Valley of South Texas. Since the two major canker diagnostic methods based on PCR and immunoassay are either unable to distinguish *Xcc* A^w^ from *Xcc* A or fail to detect *Xcc* A^w^, the verification of the causal agent of the canker detected in the Rio Grande Valley needs to be conducted in a lab, which requires multiples steps, including PCR and Sanger sequencing. This study was initiated to develop a quick and easy detection method specific to *Xcc* A^w^ that can be deployed in the field. Since Rigano et al. [24] confirmed the effectiveness of the LAMP method (CBC-LAMP) for the detection of *Xcc* and due to the easy accessibility of the LAMP technique and the reagents required for the LAMP reaction, we also adopted the LAMP technique to develop a LAMP assay specific to *Xcc* A^w^. Although this study showed that the LAMP-A^w^ assay was less sensitive than the qPCR-based method, it can detect *Xcc* A^w^ in ~30 min in a sample that has as low as ~4.5 Log_10_ copies of targets per µL, which is much faster than the qPCR-based method. Increasing the input DNA amount fivefold improved the LAMP-A^w^ assay’s sensitivity, allowing the detection of samples with approximately 3.4 Log_10_ target molecules per µL. In addition, with the increased amount of input DNA in the LAMP-A^w^ reaction, the canker detection efficiency of the LAMP-A^w^ assay using the field samples with clear canker lesions became comparable not only to that of CBC-LAMP but also to that of qPCR. These data suggested that although LAMP-A^w^ will not be a suitable method to diagnose the citrus canker caused by *Xcc* A^w^, it can successfully detect *Xcc* A^w^ in the leaves with well-established canker lesions.

In this study, we showed that the LAMP-A^w^ assay can be used in the field using a lateral flow immunoassay system as a post-detection step, which allowed the visual confirmation of the LAMP-A^w^ results on a portable lateral flow immunoassay cassette. Although 0.8% NaOH can eliminate the sample-boiling step during the crude DNA extract preparation in the field, we observed the inhibitory effect of 0.8% NaOH on the LAMP-A^w^ reaction, which required sample dilution prior to running the LAMP-A^w^ reaction to overcome the inhibitory effect. However, this additional sample dilution step has increased the complexity of the whole assay procedure and uncertainty regarding the interpretation of the in-field LAMP-A^w^ assay results. Therefore, it is more advisable to adopt the sample-boiling step using sterilized water for the LAMP-A^w^ assay in the field. In addition, the data showed that the lateral flow assay for the visual detection of the LAMP-A^w^ results was less sensitive than the LAMP-A^w^ assay conducted on the real-time PCR. Therefore, the study suggested that it is necessary to re-test the samples that tested negative for *Xcc* A^w^ on the lateral flow assay cassette in the lab either by qPCR or LAMP-A^w^ conducted on the real-time PCR system.

Although the data in the study revealed a limitation of the field applicability of the LAMP-A^w^ assay, we have confirmed that using the LAMP-A^w^ assay on a real-time system provided an efficient and novel method to distinguish *Xcc* A^w^ from *Xcc* A on the symptomatic leaves. To our knowledge, it is the first report of a molecular detection method specific to *Xcc* strain A^w^.

## 4. Materials and Methods

### 4.1. Sample Collection and Crude DNA Extract Preparation

As citrus canker is a quarantined disease, several restrictions existed in collecting the samples from canker-infected trees. USDA-APHIS-PPQ surveyors visited and collected symptomatic leaves. The crude DNA extracts that were used for qPCR and the LAMP-A^w^ reaction conducted on a real-time system were prepared from small sections of the leaves with canker symptoms by placing them into a microfuge tube filled with 200 µL of a nuclease-free water followed by boiling the sample tubes for 10 min. The sample tubes were centrifuged for 5 min at 10,000 rpm from which the supernatant was transferred into a new microfuge tube and kept at −20 °C. The LAMP-A^w^ reaction in the study was conducted with 1 µL of the crude DNA extracts prepared with nuclease-free water, except for the samples in Table 1 where the amount of input DNA was indicated in the table footnote.

The crude DNA extracts used for the LAMP-A^w^ assay coupled with lateral flow immunoassay were prepared from 100 mg of symptomatic leaf sections placed in a sample mesh bag (Agdia, Elkhart, IN, USA) where 500 µL of 0.8% NaOH or AMP1 buffer (Agdia) was added. Then, the sample bags were crushed with a blunt object. Approximately 100–150 µL of crude leaf extracts were collected into a microfuge tube that was kept at −20 °C. For the LAMP-A^w^ assay coupled with a lateral flow assay system, the crude extracts prepared with 0.8% NaOH were diluted 10-fold with nuclease-free water from which 5 µL of diluted crude extract was used for the LAMP-A^w^ reaction.

### 4.2. LAMP Primer Design

A set of six LAMP primers specific to *Xcc* A^w^ was designed based on the *avrGF1* gene of *Xcc* A^w^ (DQ275469.1) using the NEB LAMP primer design tool (https://lamp.neb.com/#!/, accessed on 24 October 2024) (Figure 1). The overall workflow to develop a LAMP assay specific to *Xcc* A^w^ was summarized in Supplementary Figure S1.

### 4.3. Real-Time PCR

The canker status of all samples used in the study was first verified by the qPCR using the primer/probe set that targets the *Xcc pthA* gene, VM3 (5′-GCA TTT GAT GAC GCC ATG AC-3′), R-Xac-R (5′-CAC TCT GCG AAA GAG CTG TAA CA-3′) and a probe (5′-TCG GGA TGA GCA GGC ACG GG-1-3′), as shown in the USDA work instruction WI-B-T-1-52. The qPCR mixture is composed of 1 µL crude DNA extract, 0.4 µM VM3/R-Xac-R, 0.2 µM probe, 6 mM MgCl_2_, 0.3 mM dNTPs, 1.25 U Platinum Taq DNA polymerase (ThermoFisher, Waltham, MA, USA) and 1x Platinum Taq DNA polymerase buffer in 25 µL of reaction volume. The qPCR was conducted on a CFX96 real-time system (BioRad, Hercules, CA, USA) following one cycle of the initial denaturation at 95 °C for 3 min, followed by 40 cycles of 95 °C for 10 s and 60 °C for 30 s.

The qPCR conducted in the study for the comparison with the LAMP reaction was conducted with the LAMP primer set, F3 and B3 (Figure 1), using SsoAdvanced Universal SYBR Green Supermix (BioRad). The qPCR mix contained 1 µL crude DNA extract, 0.4 µM F3/B3 and 10 µL of SsoAdvanced Universal SYBR green supermix in 20 µL of reaction volume. The qPCR was conducted on a CFX96 real-time system following one cycle of initial denaturation at 95 °C for 3 min, then 40 cycles of 95 °C for 10 s and 60 °C for 30 s, followed by a melt curve analysis with a default setting.

### 4.4. Optimization of the LAMP Assay

The optimization of the LAMP-A^w^ assay was performed on CFX96 real-time system using the LAMP protocol for Bst2.0 WarmStart DNA polymerase (NEB) as a guideline. The optimization was conducted in three steps: (1) optimization of LAMP reaction temperature by conducting a gradient LAMP assay with the temperature range from 50 °C to 68 °C using a *Xcc* A^w^ positive DNA sample. The LAMP reaction mixture was prepared following the LAMP protocol for Bst2.0 WarmStart polymerase (NEB). The fluorescence signal reading was conducted every 30 s (i.e., one LAMP cycle = 30 s), which was continued up to 90 cycles; (2) optimization of MgSO_4_ by conducting a LAMP assay with 4 mM, 5 mM, 5.5 mM, 6 mM MgSO_4_ at 68 °C for up to 90 cycles (one LAMP cycle = 30 s); and (3) optimization of primer concentration by setting up the LAMP reaction with different molar concentrations of LAMP primers (FIP/BIP: F3/B3: LF/BF). The molar ratios of the LAMP primers used for the optimization were (a) FIP/BIP: F3/B3: LF/LB = 1.6 µM: 0.4 µM: 0.2 µM, (b) FIP/BIP: F3/B3: LF/LB = 1.6 µM: 0.4 µM: 0.4µM, (c) FIP/BIP: F3/B3: LF/LB = 1.6 µM: 0.4 µM: 0.8 µM, and (d) FIP/BIP: F3/B3: LF/LB = 1.6 µM: 0.2 µM: 0.4 µM. From these optimization processes, 68 °C, 5.5 mM MgSO_4_ and the primer molar ratio of FIP/BIP: 1.6 µM, F3/B3: 0.4 µM and LF/BF: 0.8 µM were selected as optimal for the LAMP-A^w^ assay used in the study.

### 4.5. Lateral Flow Immunoassay

The LAMP-A^w^ reaction for the lateral flow immunoassay using the NALF-D cassette (DCNovations) was conducted in a conventional PCR at 68 °C for 45 min using two modified loop primers, Biotin-labeled LF and DIG-labeled LB. After the completion of the LAMP-A^w^ reaction, 5 µL of the LAMP reaction mixture was mixed with 70 µL of the running buffer (DCNovations), which was loaded to the sample port on the NALF-D cassette for post-amplification detection.

## 5. Conclusions

In this study, we developed a LAMP assay that can specifically detect *Xcc* A^w^ without the cross-reactivity with *Xcc* A and verified the field applicability of the LAMP-A^w^ assay coupled with a lateral flow immunoassay cassette allowing the visual confirmation of the test results. While the LAMP-A^w^ assay demonstrated limitations in field use due to a reduced sensitivity with low bacterial titers, it also showed significant advantages when coupled with a lateral flow assay. This combination provides a practical alternative to lab-based methods requiring a real-time PCR system: (1) the LAMP reaction can be run in the field on a portable heat block that can be connected to the vehicle 12 V outlet; (2) the test result can be obtained in the field in about 45 min, including the crude DNA extract preparation; and (3) combining the LAMP-A^w^ assay together with the CBC-LAMP assay [24] allows rapid in-field identification of bacterial strains (*Xcc* A vs. *Xcc* A^w^), enabling timely implementation of appropriate disease management strategies.

## Figures and Tables

**Table 1:** Evaluation of *Xcc* A^w^ detection efficiency of the LAMP-A^w^ assay compared to qPCR and CBC-LAMP assay that targets the *Xcc pthA* gene.

Sample ^a^	Ct Value (qPCR) ^b^	LAMP-A^w^ Ct Value ^c^	CBC-LAMP Ct Value ^d^
C-201-3	20.66	18.41	18.05
C-201-52	28.32	29.08	24.28
C-201-53	29.96	30.98	25.75
C-201-54	29.24	30.68	24.99
C-201-55	27.39	28.45	23.59
C-201-56	31.38	31.91	27.16
C-201-57	32.46	31.79	28.19
C-201-58	28.92	30.79	23.75
C-201-59	29.10	30.35	26.87
C-201-61	18.97	18.97	17.13
C-201-62	20.82	21.29	18.32
C-137	23.78	24.32	20.77
C-232	20.04	21.03	17.63
C-229	28.36	29.81	24.21
C-149	N/A	N/A	N/A
C-156	N/A	N/A	N/A
C-160	N/A	N/A	N/A
C-163	N/A	N/A	N/A
C-164	N/A	N/A	N/A

^a^ The crude DNA extracts were prepared by soaking the canker lesion in sterilized water followed by boiling for 10 min. ^b^ qPCR was conducted with the LAMP-A^w^ F3/B3 primer set using 1 µL of crude DNA extracts. ^c^ LAMP-A^w^ reaction was conducted with 5 µL of crude DNA extracts. One LAMP-A^w^ cycle equals 30 s. ^d^ CBC-LAMP reaction (15) that targets the *pthA* gene of *Xcc* was conducted with 1 µL of crude DNA extracts. One CBC-LAMP cycle equals 30 s.

## Data Availability

Data is contained within the article or Supplementary Material.

## Supplementary Materials

The following supporting information can be downloaded at: https://www.mdpi.com/article/10.3390/ijms252111590/s1.
