# Supplementary material for: Loop-Mediated Isothermal Amplification Assay for the Detection of Citrus Canker Causing Bacterial Variant, Xanthomonas citri pv. citri Aw Strain
Source: Int J Mol Sci. 2024 Oct 29;25(21):11590. doi: 10.3390/ijms252111590 (PMC11546398; doi:10.3390/ijms252111590)
Supplement: Supplementary file 1 [file ijms-25-11590-s001.zip › ijms-3263904-supplementary.pdf]

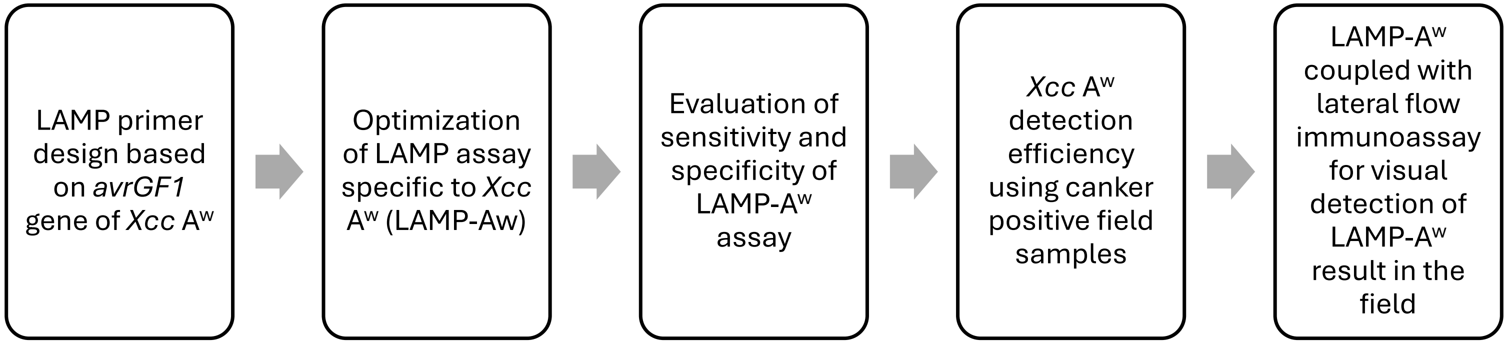

Supplementary Figure S1. The workflow for the development of the LAMP-A^w assay coupled with lateral flow immunoassay system.
